# CD203c is expressed by human fetal hepatoblasts and distinguishes subsets of hepatoblastoma

**DOI:** 10.3389/fonc.2023.927852

**Published:** 2023-02-09

**Authors:** Marcus O. Muench, Marina E. Fomin, Alan G. Gutierrez, Dolores López-Terrada, Renata Gilfanova, Christopher Nosworthy, Ashley I. Beyer, Gregory Ostolaza, Dina Kats, Kevin L. Matlock, Stefano Cairo, Charles Keller

**Affiliations:** ^1^ Vitalant Research Institute, San Francisco, CA, United States; ^2^ Department of Laboratory Medicine, University of California, San Francisco, San Francisco, CA, United States; ^3^ Department of Pathology and Immunology, Baylor College of Medicine, Houston, TX, United States; ^4^ Texas Children’s Cancer Center, Texas Children’s Hospital, Houston, TX, United States; ^5^ Pediatric Cancer Biology, Children’s Cancer Therapy Development Institute, Beaverton, OR, United States; ^6^ Omics Data Automation, Beaverton, OR, United States; ^7^ Research and Development Unit, XenTech, Evry, France

**Keywords:** fetal cells, antigens, hepatology, hepatoblasts, endothelial cells, leukocytes, CD203c, hepatoblastoma

## Abstract

**Background & Aims:**

Hepatocytic cells found during prenatal development have unique features compared to their adult counterparts, and are believed to be the precursors of pediatric hepatoblastoma. The cell-surface phenotype of hepatoblasts and hepatoblastoma cell lines was evaluated to discover new markers of these cells and gain insight into the development of hepatocytic cells and the phenotypes and origins of hepatoblastoma.

**Methods:**

Human midgestation livers and four pediatric hepatoblastoma cell lines were screened using flow cytometry. Expression of over 300 antigens was evaluated on hepatoblasts defined by their expression of CD326 (EpCAM) and CD14. Also analyzed were hematopoietic cells, expressing CD45, and liver sinusoidal-endothelial cells (LSECs), expressing CD14 but lacking CD45 expression. Select antigens were further examined by fluorescence immunomicroscopy of fetal liver sections. Antigen expression was also confirmed on cultured cells by both methods. Gene expression analysis by liver cells, 6 hepatoblastoma cell lines, and hepatoblastoma cells was performed. Immunohistochemistry was used to evaluate CD203c, CD326, and cytokeratin-19 expression on three hepatoblastoma tumors.

**Results:**

Antibody screening identified many cell surface markers commonly or divergently expressed by hematopoietic cells, LSECs, and hepatoblasts. Thirteen novel markers expressed on fetal hepatoblasts were identified including ectonucleotide pyrophosphatase/phosphodiesterase family member 3 (ENPP-3/CD203c), which was found to be expressed by hepatoblasts with widespread expression in the parenchyma of the fetal liver. In culture CD203c^+^CD326^++^ cells resembled hepatocytic cells with coexpression of albumin and cytokeratin-19 confirming a hepatoblast phenotype. CD203c expression declined rapidly in culture whereas the loss of CD326 was not as pronounced. CD203c and CD326 were co-expressed on a subset of hepatoblastoma cell lines and hepatoblastomas with an embryonal pattern.

**Conclusions:**

CD203c is expressed on hepatoblasts and may play a role in purinergic signaling in the developing liver. Hepatoblastoma cell lines were found to consist of two broad phenotypes consisting of a cholangiocyte-like phenotype that expressed CD203c and CD326 and a hepatocyte-like phenotype with diminished expression of these markers. CD203c was expressed by some hepatoblastoma tumors and may represent a marker of a less differentiated embryonal component.

## Introduction

Hepatoblasts are immature hepatocytic precursors capable of differentiating into hepatocytes or cholangiocytes. Hepatoblasts exist during human fetal development alongside a diversity of other cell types owing to the liver being a major hematopoietic tissue during much of the first half of gestation (1, 2). The presence of fetal hepatocytes, cholangiocytes, hematopoietic cells, endothelial cells, mesenchymal stromal cells, and stellate cells in the developing liver has made identification and isolation of a fetal hepatoblast difficult as many markers expressed by these cells are likely to be also expressed by other cell types as well. Indeed, a full molecular characterization of human hepatoblasts is still lacking as is an understanding of the diversity that exists among the subsets of stem cells and progenitors collectively referred to as hepatoblasts at different stages of development.

Currently, the phenotypic identification and isolation of hepatoblasts is best performed using multiple cell-surface markers based on both negative and positive expression patterns. During fetal development CD326 – epithelial cell adhesion molecule (EpCAM) – is expressed by hepatocytic stem cells capable to differentiate into albumin-expressing hepatocytes (3). Additionally, cultured fetal liver cells produce CD326^+^ progenitors that can give rise to hepatic colonies (4). In the adult liver, CD326 expression is found on biliary cells (5) and a rare subset of bipotent stem cells and lineage-skewed progenitors (3, 6–8). However, CD326 is widely expressed on parenchymal cells in the fetal liver and, thus, does not solely define a discrete subpopulation of stem cells (6, 9). CD326 is also expressed by many other cell types in the fetal liver at different intensities, so CD326 expression alone does not mark hepatocytic cell lineages. Fetal hepatoblasts have been shown to express various markers also expressed by adult hepatocytes including CD24, CD26, and CD49f (9). Markers that have been reported to be expressed by the subset of hepatocytic precursors believed to represent stem cells include CD56, CD90, CD117, CD133, and CUB domain-containing protein 1 (3, 10–13). Cytoplasmic expression of cytokeratin (CK)19 also identifies bipotent hepatocytic stem cells (3, 14), but cannot be used to isolate living cells.

The uniqueness of fetal hepatoblasts compared to adult parenchymal cells is not only of interest to the study of liver development but also of particular interest to the classification and treatment of pediatric hepatoblastoma. Hepatoblastoma is a paradigm for embryonal cancers – those in which early life (prenatal) factors drive the initiation and biology of the malignancy after birth and later in life. Hepatoblastoma is the most common primary liver tumor diagnosed in children, with approximately 1.76 cases per million persons each year in the United States (15). Hepatoblastoma incidence has risen by about 4% between 1992 and 2004, more so than any other childhood cancer, with increasing rates of prematurity as an implicated cause (16). While the survival rate for patients whose tumor can be surgically removed is 75-80% at a median of 8 years (17), the treatment options for the 1 in 5 children with unresectable or metastatic disease are extremely limited. To address this issue, scientists and clinicians have pursued non-chemotherapeutic treatments that target the molecular mechanisms of hepatoblastoma proliferation and metastasis. To date, no targeted agents have been identified and no hepatoblastoma specific biology-driven therapeutics are being investigated in clinical trials. This paucity of targeted therapies can be traced to how few cell lines or mouse models of hepatoblastoma have been reported thus far (18), and how little is known of the hepatoblastoma cell(s) of origin.

In this study, we have used antibody screening using flow cytometry to determine the expression of 332 antigens on fetal hepatoblasts. This analysis revealed the expression of novel antigens on these cells including ectonucleotide pyrophosphatase/phosphodiesterase member 3 (CD203c/ENPP3). CD203c belongs to a family of ectoenzymes that share a phosphodiesterase domain and some, including CD203c, are involved in hydrolysis of extracellular nucleotides (19). The expression of CD203c was evaluated on liver cells of different gestational ages and in culture. Hepatoblastoma cell lines were also evaluated using a panel of markers selected for either their consistent or differential expression on normal hepatoblasts to phenotypically characterize these cells to gain insight into the origin(s) of these cancers and potential therapeutic targets.

## Results

### Antibody array analysis of midgestation fetal liver cells

Our group has used expression of CD14, in conjunction with CD326, to distinguish hepatoblasts and liver sinusoidal endothelial cells (LSECs) among fetal liver cells lacking the blood cell marker CD45 (9, 20). Using this staining scheme, antibody screening was performed to analyze the expression of a wide array of cell surface antigens on hepatoblasts as well as other fetal liver cell-populations as described in a the following dataset article (21). Fetal liver cells (21 weeks’ gestation) were first depleted of CD235a^+^ erythrocytes by immunomagnetic bead depletion. To define the non-hematopoietic compartment of liver cells, cells were stained with CD45 as well as labeled CD235a antibody to remove residual red cells by gating. The diverse population of hematopoietic cells comprised 71.2% of the liver cells and were analyzed for marker expression as a group. Among the CD45^-^CD235a^-^ non-hematopoietic cells, two additional groups were defined: CD14^++^ LSECs and CD14^low^CD326^++^ hepatoblasts.

**Figure 1:**
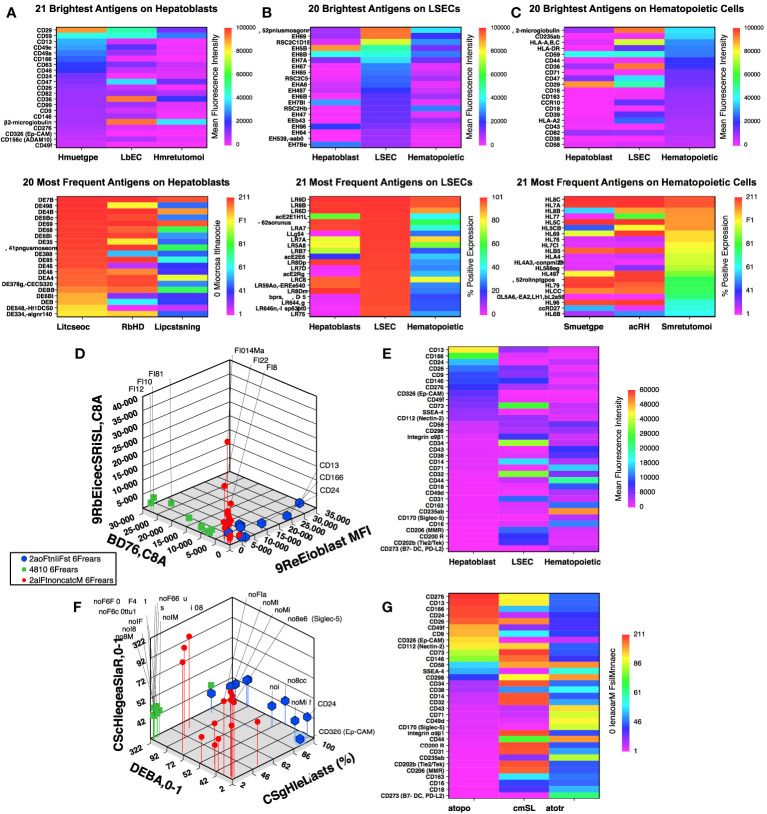
Flow cytometric array analysis of human fetal liver. Frequency and intensity of antigen expression on hepatoblasts **(A)**, LSECs **(B)**, and hematopoietic **(C)** cell populations from midgestation liver. Further description of the gated cell populations can be found in (21). Comparison of mean fluorescence intensity **(D)** and frequency of expression **(F)** of 33 selected antigens (see text) using 3-dimensional scatter plots. Corresponding heat maps for mean fluorescence intensity **(E)** and frequency of expression **(G)** for the 33 antigens are shown sorted based on antigen expression by hepatoblasts.

The hepatoblast population was evaluated for the 21 brightest – highest mean fluorescence intensity – and 20 most abundantly expressed markers (**Figure 1A**). The expression of these markers on LSECs and hematopoietic cells are shown alongside the hepatoblast markers with the same analyses performed for the brightest and most abundant LSEC (**Figure 1B**) and hematopoietic (**Figure 1C**) markers. A number of widely expressed antigen such as class I major histocompatibility associated antigens (HLA-A,B,C and β2-microglobulin), CD47, and CD59 are listed in all groups. To identify markers with more distinctive expression patterns among the three cell populations, the 51 antigens represented in **Figures 1A–C** were examined and any antigen found expressed in two or more lineages eliminating from the subset. The resulting 33 antigens were compared using 3-dimensional scatter plots showing mean fluorescence intensity (**Figures 1D, E**) and frequency of expression (**Figures 1F, G**). The identity of the markers showing the brightest or most abundant staining restricted to either hepatoblasts, LSECs or hematopoietic cells are indicated on the scatterplots. Heat maps for the 33 antigens were arranged based on their expression on hepatoblasts (**Figures 1E, G**).

Among the antigens identified from these analyses with the most frequently and high expression on hepatoblasts were markers previously reported to be expressed on fetal hepatoblasts: CD9, CD13, CD24, CD26, CD29, CD46, CD49a, CD49e, and CD49f (9, 22, 23). The expression of these markers on CD14^low^CD326^++^ hepatoblasts are shown in an analysis that has been uploaded to a data repository as described in (21). CD36 expression was observed on hepatoblasts, although not at levels as high as on LSECs. Nonetheless, confirming in the prenatal liver CD36 expression as documented on adult hepatocytes (24, 25). Also among these recognized hepatocytic cell markers was CD326 (EpCAM) (3, 4, 7) which, to be noted, was co-stained by the same antibody clone in two colors in our screening.

**Figure 2:**
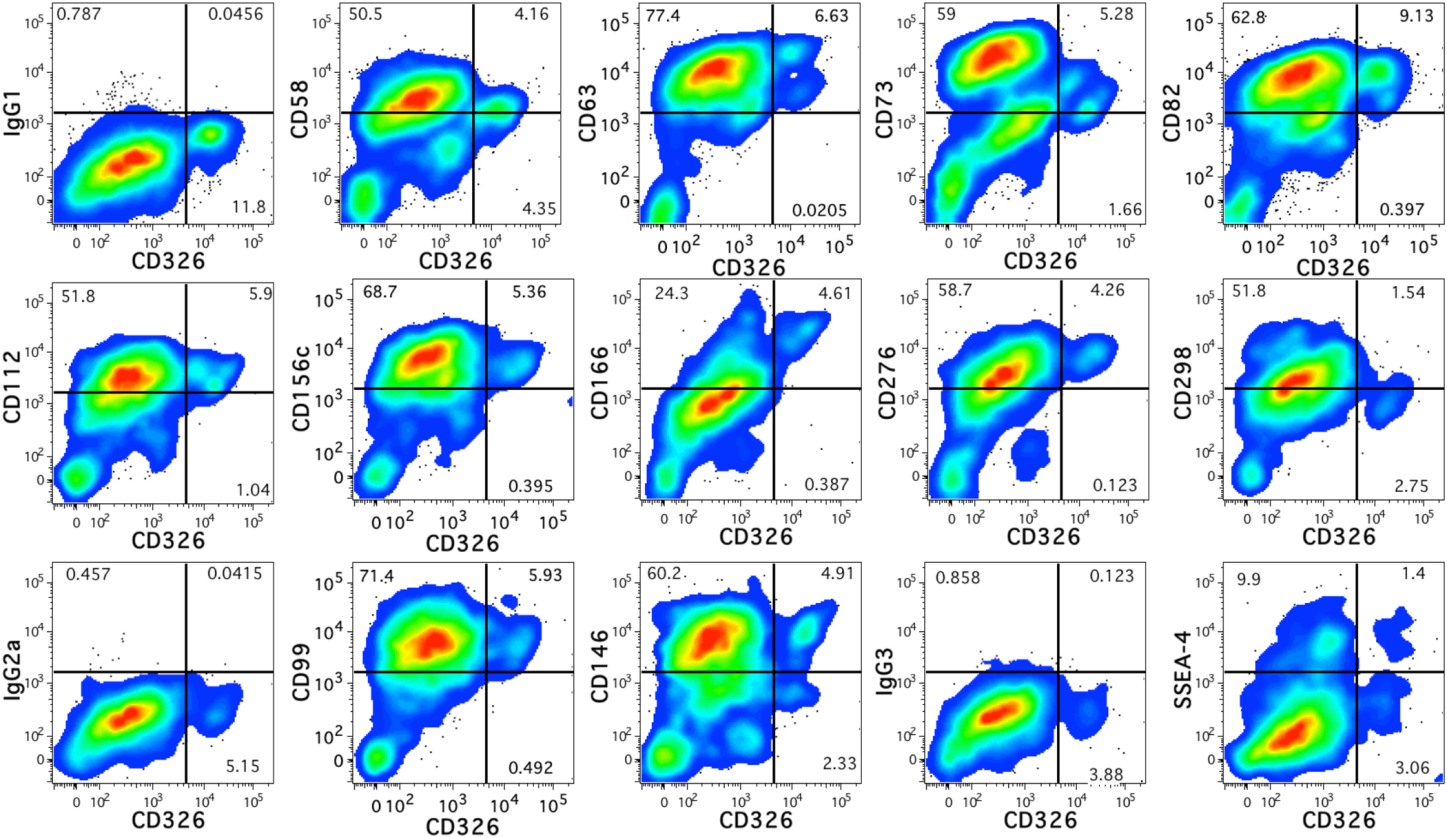
Antigen expression on fetal hepatoblasts. Novel antigens expressed by human fetal hepatoblasts identified by high levels of CD326 staining. The relevant isotype controls are shown preceding plots showing the antigen stains beginning with IgG1 at the top-left.

The remaining twelve novel markers that were identified are shown in **Figure 2** in relation to CD326 expression. These include CD58 (lymphocyte function-associated antigen 3 or LFA-3) which can bind CD2 to stimulate T-cells (26) and CD166 (activated leukocyte cell adhesion molecule or ALCAM), which can participate in homophilic interactions as well as act as a ligand for CD6 (27). CD63 and CD82, members of the tetraspanin family of proteins known for multiple protein interactions and cellular functions (28, 29), were highly expressed. CD112 (Nectin-2), a component of adherens junctions (30) that also acts as a regulator T- and NK-cell cytotoxicity (31) was also observed. CD276 (B7-H3) is an immune checkpoint molecule (32) was uniformly expressed by CD14^low^CD326^++^ cells. CD73 (5′-nucleotidase or NT5E), CD146 (melanoma cell adhesion molecule or MCAM), and stage-specific embryonic antigen 4 (SSEA-4), markers associated with mesenchymal stromal cells (33), were also expressed by hepatoblasts. Other proteins observed on the hepatoblast population were CD99 (34), the protease CD156c, and CD298 (sodium/potassium-transporting ATPase subunit beta-3 or ATP1B3), which regulates Na^+^ and K^+^ ions across the plasma membrane (35).

### CD203c is expressed by fetal hepatoblasts

**Figure 3:**
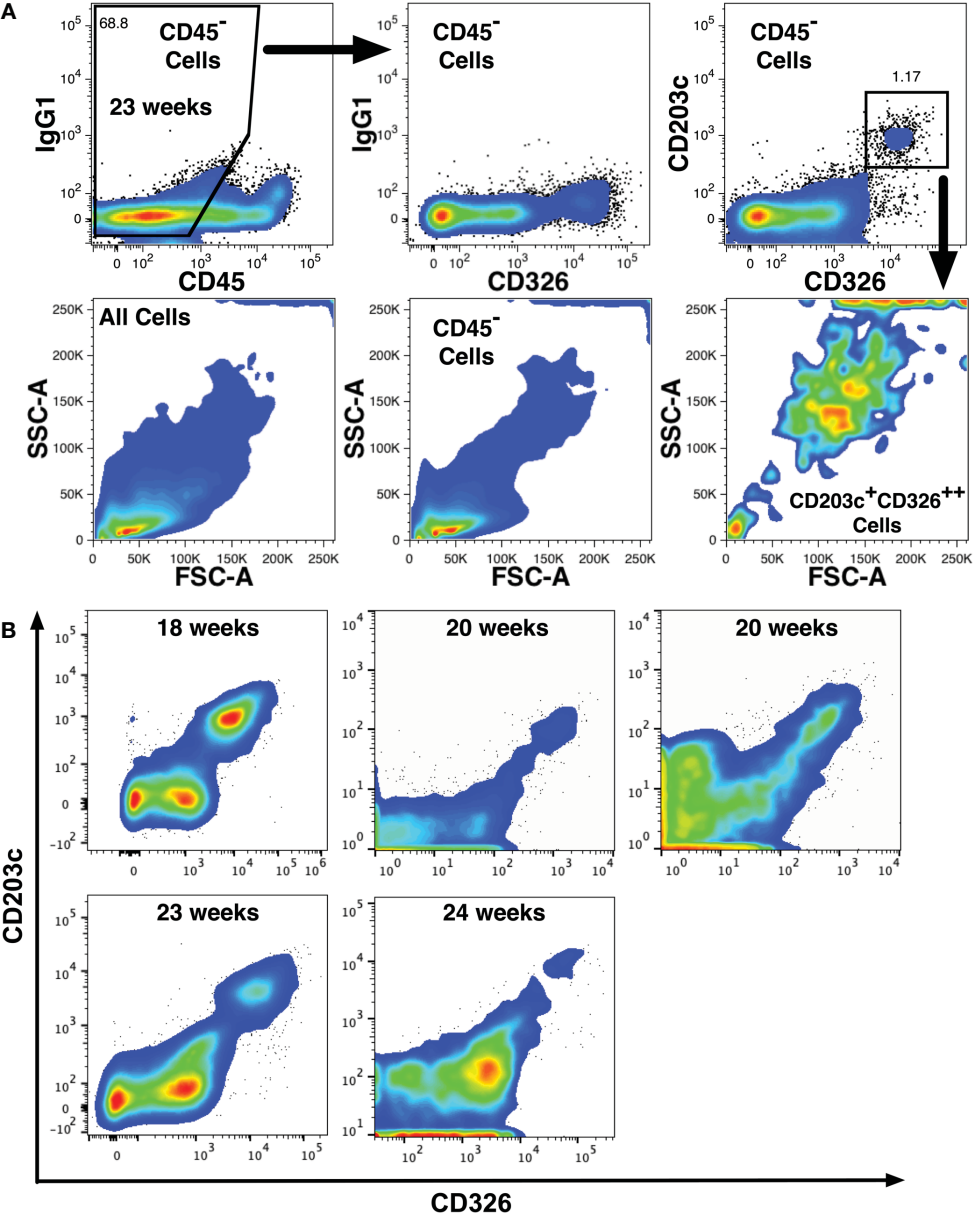
CD203c expression on fetal hepatoblasts. **(A)** CD203c expression by 23 weeks’ gestation liver cells lacking CD45 expression but expressing high levels CD326 (CD326^++^). Light scatter properties of the cell populations shown in the top row are shown in the second row and indicate that CD203c^+^CD326^++^ cells are large and have a complex structure. **(B)** Expression of CD203c and CD326 on CD45^-^ liver cells at different gestational ages from 5 additional liver specimens.

Additional phenotype screening was performed using smaller antibody panels to verify findings made with the full panel. These screens identified a thirteenth novel marker, CD203c (ectonucleotide pyrophosphatase/phosphodiesterase family member 3) that is expressed on CD14^low^CD326^++^ cells (**Figure 3**). CD203c is a recognized marker of mast cells and basophils (36, 37), which have been detected among fetal liver cell preparations enriched for hematopoietic cells (38). Analysis of CD45^-^ fetal liver cells also indicated widespread CD203c expression on CD326^++^ hepatoblasts (**Figure 3A**). The light-scatter profile of the CD203c^+^CD326^++^ cells indicated that these cells were mostly large, complex cells as expected for hepatoblasts (9). CD203c expression was confirmed with 5 additional midgestation liver specimens that expressed CD203c on a median 93% (range 71.8 - 100%) of CD326^++^ cells (**Figure 3B**).

**Figure 4:**
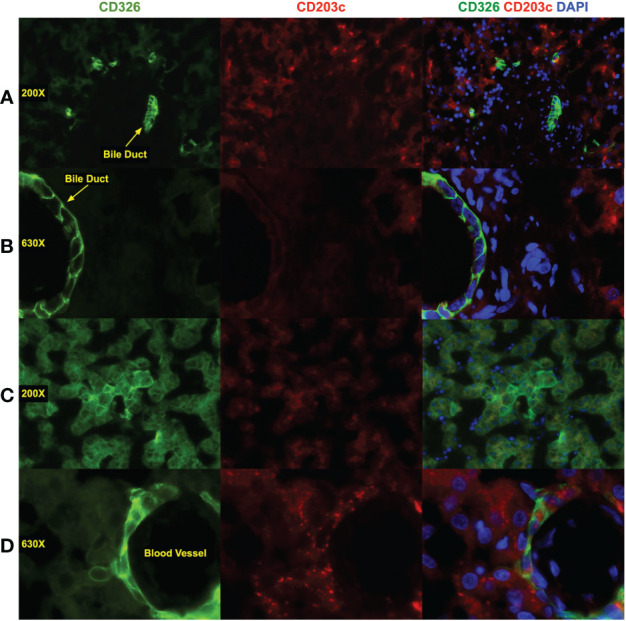
Immunofluorescence staining of fetal liver. Liver was fixed and frozen sections prepared that were stained with antibodies against CD326 (AlexaFluor 488, green), CD203c (AlexaFluor 568, red), and DAPI to stain nuclei (blue). CD203c was stained using clone 97A6 **(A, B)** and clone FR3-16A11 **(C, D)**. Images were photographed using a Leica CTR6500 microscope with 200X **(A, C)** and 630X **(B, D)** magnification. Image analysis was performed using Media Cybernetics *In Vivo* and Apple iPhoto software. Color channels were enhanced individually, with uniform application of all processes, before generating composite, shown in the composite photomicrographs in the left column.

Epifluorescence microscopy was used to evaluate CD203c and CD326 staining on midgestation liver sections (**Figure 4**). Overall, expression levels of CD326 tended to be higher than CD203c. Two antibodies were tested, clone 97A6 (**Figures 4A, B**) and clone FR3-16A11 (**Figures 4C, D**). Low expression of CD203c and CD326 was observed on hepatoblasts in the parenchyma seen surrounding a portal tract in **Figure 4A**. Mesenchymal cells in the portal tract were negative for both markers. Although cholangiocytes from a bile duct observed in the portal tract were brightly stained with CD326, little or no CD203c expression was observed. This same pattern of expression was observed at higher magnification of another bile duct (**Figure 4B**). Staining of a section of parenchyma with the clone FR3-16A11 antibody also showed co-expression of CD203c and CD326 on hepatoblasts. The more intense staining of CD326 observed surrounding central veins, portal tracts, and on cholangiocytes was not observed with CD203c.

**Figure 5:**
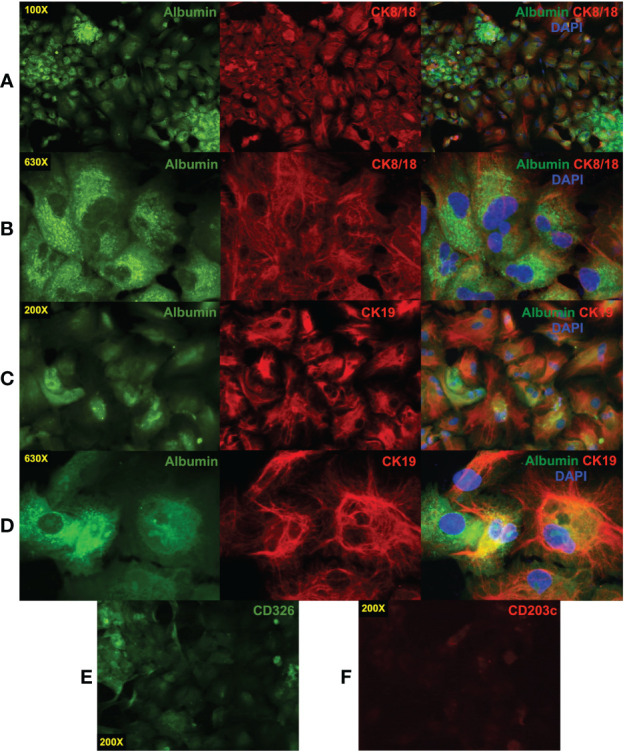
Immunofluorescence staining of cultured CD203c^+^CD326^++^ cells. CD203c^+^CD326^++^ cells were isolated by cell sorting and cultured for 11 days in William’s E medium. The cultured cells have a typical morphology of hepatocytes in tissue culture that express albumin and cytokeratin (CK)8/18 **(A)**. Multinucleated cells are evident at high magnification (630X, **(B)**. Coexpression of the hepatoblast markers albumin and CK19 was also observed **(C, D)** as well as more polarized expression of these markers suggesting differentiation into albumin^+^ hepatocytes and CK19^+^ cholangiocytes. Low levels of CD326 **(E)** and a near absence of CD203c **(F)** expression were observed on the cultured cells. Photomicrographs were prepared as described in **Figure 4**.

CD203c^+^CD326^++^ cells were isolated and cultured to confirm their identity as hepatoblasts. After 11 days of culture, the cells had the morphology of hepatocytes and expressed albumin, CK8/18, and CK19 (**Figure 5A**). Multinucleated cells were also observed under higher magnification (**Figure 5B**). Bipotent hepatoblasts coexpress the hepatocyte marker albumin and the biliary marker CK19 (7, 39) and such coexpression was observed (**Figures 5C, D**). Cultured cells with polarized expression of albumin and CK19 indicate differentiation into the two hepatocytic cell types.

### Rapid loss of CD203c expression in culture

**Figure 6:**
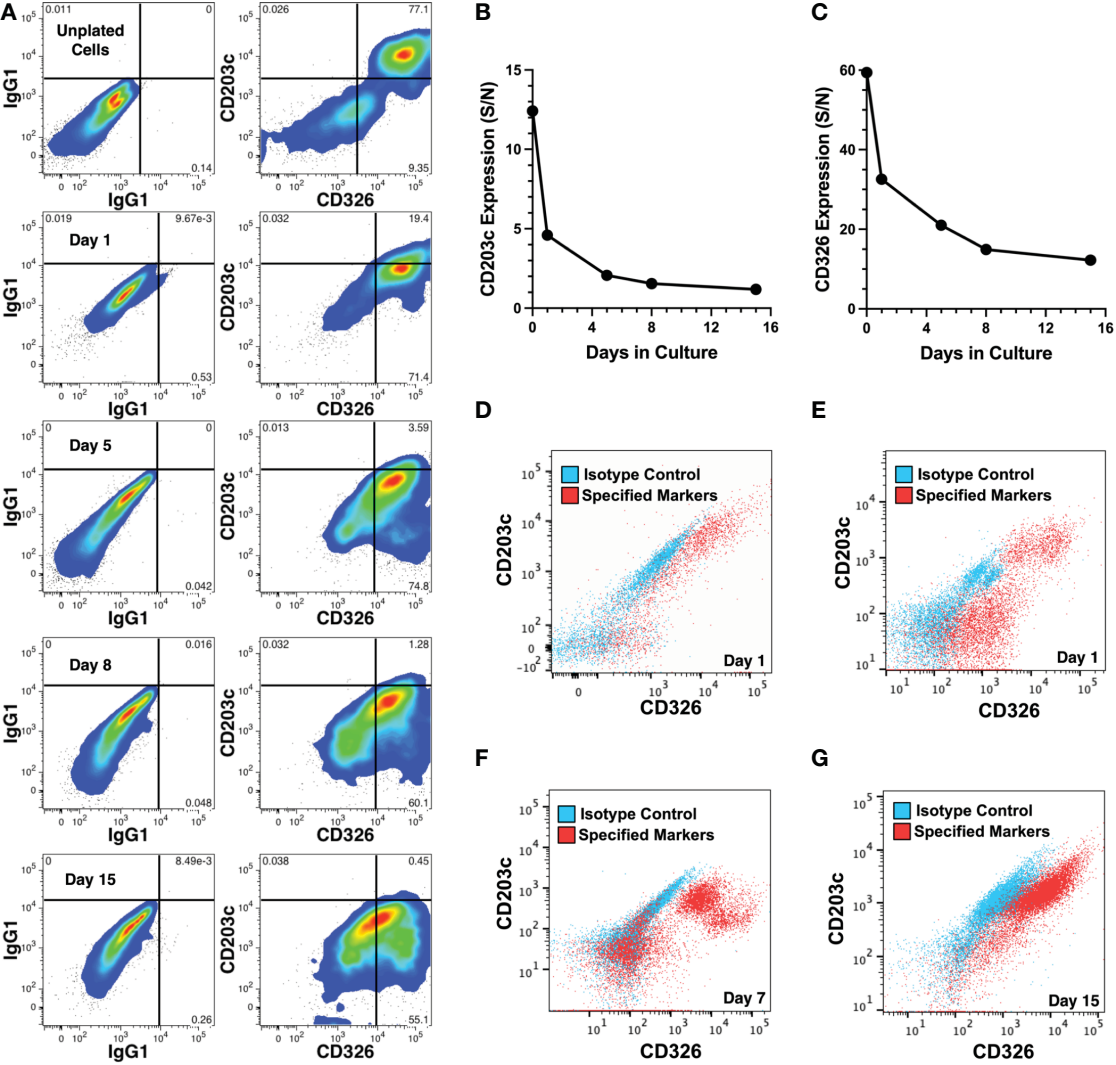
Changes in CD203c and CD326 expression during cell culture. **(A)** CD203c and CD326 expression shown on CD45^-^ liver cells from a 23 weeks’ gestation specimen stained before and 1-15 days after culture. **(B)** Expression of CD203c represented as a signal (CD203c) to noise (IgG1) (S/N) ratio of mean fluorescence intensities for the data in **(A)**. **(C)** Expression of CD326 represented as a S/N ratio of mean fluorescence intensities for the data in **(A)**. Erythrocyte depleted liver cells, 21 **(D)** and 22 **(E)** weeks’ gestation, cultured for 1 day and adherent cells analyzed for CD203c and CD326 expression on CD45^-^ cells. **(F)** Marker expression analyzed after 7 days of culture of the 22 weeks’ gestation specimen. **(G)** CD203c^+^CD326^++^ cells, isolated from an 18 weeks’ gestation liver by cell sorting, were cultured for 15 days. CD203c and CD326 expression are shown in red and background staining with isotype matched antibodies is overlaid in blue **(D–G)**. Cultures for all 4 experiments used William’s E medium.

The expression of CD326 and CD203c on cultured cells was also examined by epifluorescence microscopy which revealed that low levels of CD326 remained after 11 days (**Figure 5E**) but little to no CD203c expression was observed (**Figure 5F**). This was confirmed in another experiment using flow cytometry to quantify expression levels over 15 days of culture of erythrocyte-depleted fetal liver cells (**Figure 6A**). After only 1 day in culture, CD203c expression levels had noticeable declined. The rapid loss of CD203c expression is evident when the data are represented as a CD203c-signal to control-noise (S/N) ratio (**Figure 6B**). CD326 expression also declined in culture, but to a lesser degree than CD203c (**Figure 6C**). Partial loss of CD203c and to a lesser extent, CD326, was confirmed after 1 day of culture with two fetal liver cell preparations (**Figures 6D, E**). Notable loss of CD203c expression was observed after 7 days (**Figure 6F**) and after 15 days of culture of sorted CD203c^+^CD326^++^ cells (**Figure 6G**). It is important to note that declines in the S/N ratios occur, in part, due to the increase in autofluorescent noise resulting from cells increasing their size in culture, and not just because of a decline in marker levels.

### Hepatoblastoma tumor cells share features of two fetal liver progenitor cell populations

**Figure 7:**
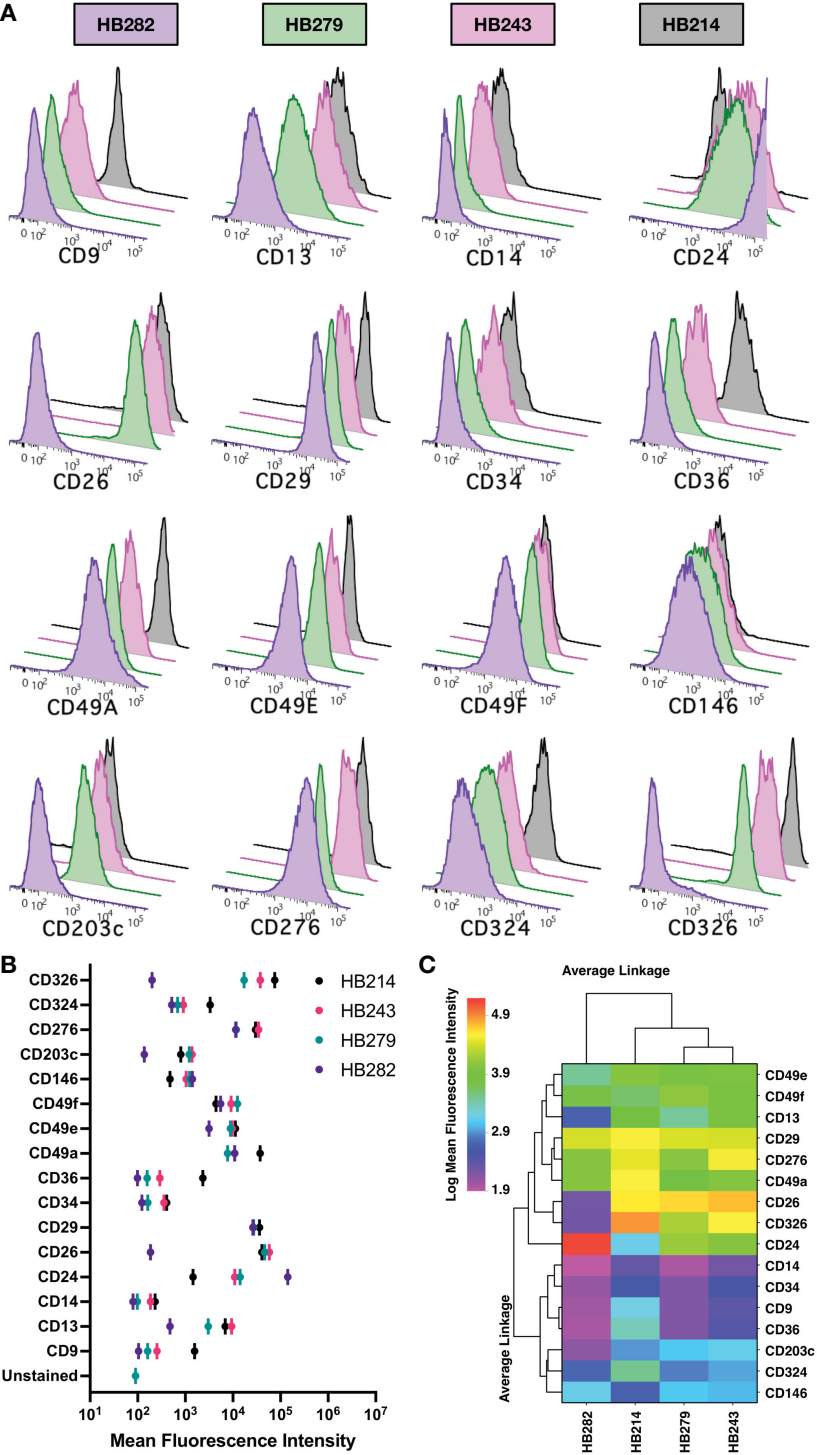
Marker expression on hepatoblastoma cell lines. **(A)** Expression of 16 markers were analyzed by flow cytometry on 4 cell lines and the relative expression data shown as histograms in overlay plots for each marker. **(B)** Mean fluorescence intensities of each marker are compared for each cell line. **(C)** The expression of the markers on cell lines is shown as a heat map ordered based on clustering using an unweighted pair-group method using arithmetic averages and Euclidian distancing. A log transformation of the mean fluorescence intensity data was performed prior to analysis to better resolve the data on the linear scale used to show heat map data.

**Figure 8:**
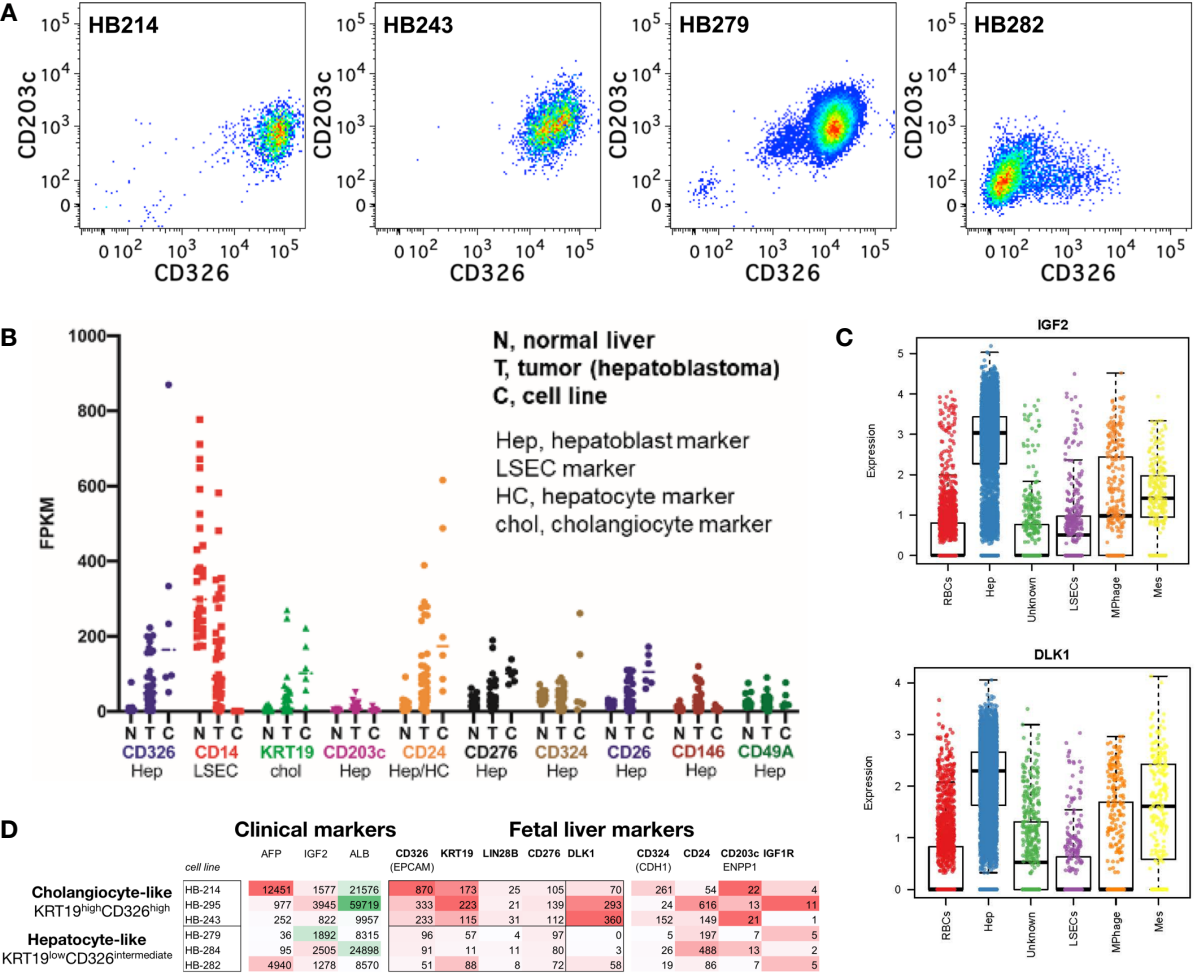
Hepatoblastoma phenocopies fetal hepatoblasts and cholangiocytes including a CD203c phenocopy. **(A)** Expression of CD326 and CD203c in hepatoblastoma cell lines. **(B)** Hepatoblastoma tumor biopsies and hepatoblastoma cell lines have reduced or absent LSEC markers (*e.g.*, CD14), bimodal cholangiocyte markers (*e.g.*, *KRT19*) and mixed/bimodal hepatoblast markers (CD326, CD24, CD324 and CD26). **(C)** Single cell sequencing of fetal liver hepatoblasts reveals cell-type selective enrichment of *IGF2* and *DLK1*, which are feasible therapeutic targets, as well as AFP (a known hepatoblastoma marker) in FPKM. **(D)** Upon closer examination of known hepatoblast markers, two phenotypes of hepatoblastoma cell lines are observed: cholangiocyte-precursor-like and hepatoblast/hepatocyte-precursor-like.

As some pediatric hepatoblastoma are believed to have a prenatal origin based upon prenatally detected onset (16), we investigated the expression of 16 markers expressed by fetal hepatoblasts using four cell lines (HB214, HB243, HB279, HB282) derived from pediatric hepatoblastoma tumors diagnosed at 31 days, 52 days, 79 days and 12 months of age, respectively (40). Mostly uniform expression of CD29, CD49a, CD49e, CD49f, CD146, CD276, and CD324 was observed (**Figures 7A, B**). On the other hand, CD14 and CD34 was generally negligible. More interesting were markers that had variable expression on the different cell lines, which included CD9, CD13, CD24, CD26, CD36, CD203c, and CD326. Linkage analysis using these markers revealed HB282 cells to differ from the other three cell lines (**Figure 7C**). HB282 cells were most notably distinguished by their very high expression levels of CD24 and lack of CD26, CD203c, and CD326 expression (**Figures 7**, **8A**).

To understand the markers and potential cellular origins/ontogeny of hepatoblastoma tumor cells, RNA expression was examined for fetal liver lineage markers from hepatoblastoma tumors (an admixture of stromal cells and tumor cells) and hepatoblastoma tumor cell lines (tumor cells alone). Tumor cells lacked LSEC markers (*e.g.*, CD14) but had bimodal hepatoblast and cholangiocyte marker expression (*e.g.*, the CK19 gene *KRT19*) (**Figure 8B**). Further examination of hepatoblastoma tumor cell line markers uncovered bimodal expression of *CD326*, *KRT19*, *LIN28B* and *CD276* dividing cell lines into two phenocopies: cholangiocyte-precursor-like (*KRT19* ^high^ *CD326* ^high^) and hepatoblast/hepatocyte-precursor-like (*KRT19* ^low^ *CD326* ^intermediate^; **Figure 8C**), akin to the normal ontogeny (1, 41). Single cell sequencing of fetal liver affirmed *DLK1* as another marker of the *KRT19* ^high^ *CD326* ^high^ hepatoblastoma phenocopy with potential clinical relevance (**Figures 8C, D**).

### CD203c expression by hepatoblastoma

**Figure 9:**
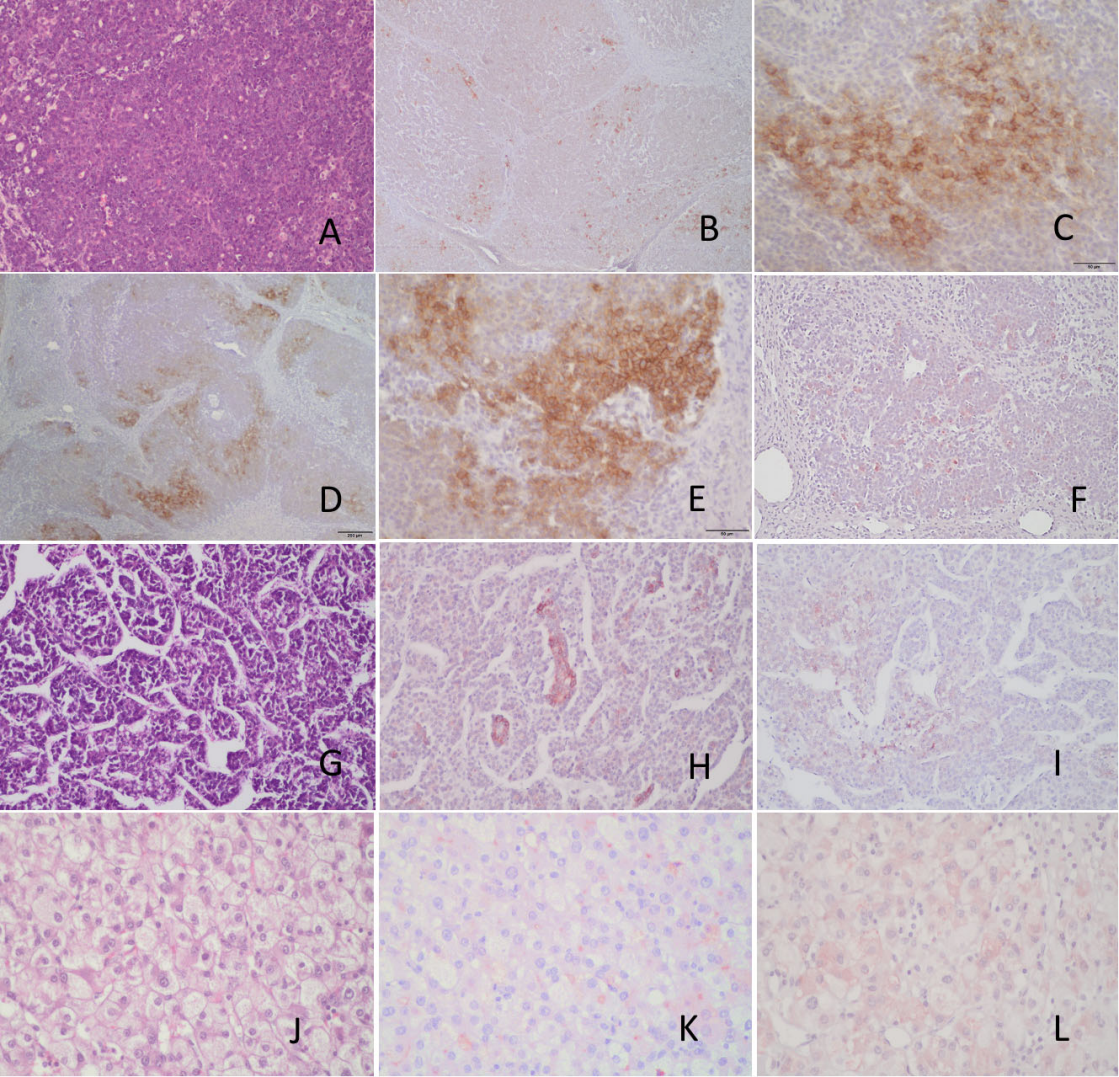
Immunohistochemical analysis of CD203c and CD326 expression by hepatoblastomas. **(A)** Epithelial hepatoblastoma diagnosed in a 18 month-old boy (stage 3, multifocal) H&E. **(B, C)** CD203 immunohistochemistry showing very focal membranous positive tumor cells. **(D, E)** CD326 immunohistochemistry demonstrating also focal positive cells (membranous) in the periphery of the embryonal type tumor nodules. **(F)** the tumor is negative for CK19. **(G)** H&E stain of another hepatoblastoma, epithelial embryonal type diagnosed in a 5 yo female (Pretext stage 4), showing focal positive CD203c cells **(H)** and very focal, weak CD326 positivity **(I)**. Pure fetal hepatoblastoma **(J)** negative for CD203c **(K)** and CD326 **(L)**. Scale bars 50μ **(C, E)** and 200μ **(D)**.

CD203c expression was examined in three cases of hepatoblastoma. In the first case, a multifocal epithelial hepatoblastoma (stage 3) with both fetal and embryonal elements (**Figure 9A**) demonstrated focal membranous CD203c expression (**Figures 9B, C**). Foci of membranous CD326 expression were also observed in the periphery of the embryonal type tumor nodules (**Figures 9D, E**). CK19 expression was not detected (**Figure 9F**). A second case of epithelial embryonal hepatoblastoma (**Figure 9G**), pre-therapy extent of the tumor (PRETEXT) stage 4 (42), also exhibited focal CD203c expression (**Figure 9H**) and very weak and focal CD326 positivity (**Figure 9I**). In contrast, a case of PRETEXT stage 3 fetal hepatoblastoma (**Figure 9J**) did not express CD203c (**Figure 9K**) or CD326 (**Figure 9L**).

## Discussion

The human liver undergoes profound changes during early development transforming from a primarily erythropoietic organ during the first half of gestation (1) into an organ comprised mostly of large hepatocytes at birth (43). Hematopoietic and mature blood elements persist in the adult liver and contribute to the important immune functions of this organ, but in adults the liver is not normally a primary hematopoietic organ (38, 44–47). From the onset of liver development until the neonatal period, fetal hepatoblasts grow and mature into hepatocytes exposed to a rapidly changing environment that includes the rise and fall of hematopoietic cells, hormonal changes, and the dramatic changes in blood flow at birth that alter the flow of nutrients and oxygenated blood (43). Given the unique nature of the prenatal liver, it is not surprising that fetal hepatocytic cells differ from their postnatal counterparts.

This study focused on the cell-surface phenotype of fetal hepatoblasts to identify antigens that may differentiate subsets of these cells or contribute to the unique biology of these cells. Flow cytometric screening revealed 13 antigens (**Figures 2** and **3**) of interest. Of these, CD203c, which is expressed by most fetal liver parenchymal cells, was analyzed in greatest detail. The ectoenzyme CD203c – ENPP3 – was initially recognized as a marker of basophils and tissue mast cells (36, 37). CD203c catalyzes the hydrolysis of nucleotide phosphate diester bonds, preferentially adenosine triphosphate, but has low specificity and may degrade other potential substrates such as nucleotide sugars, diadenosine polyphosphates, and the dinucleotide diguanosine-tetraphosphate (19). The capacity for hydrolysis of adenosine triphosphate points to a role for CD203c in regulating purinergic signaling in the developing liver (48, 49). Additionally, CD73 expression on fetal hepatoblasts and CD39 expression by LSECs and hematopoietic cells likely also contribute to the regulation of purinergic signaling in the fetal liver.

The expression of CD203c by hepatocytic cells in the liver is not widely recognized. In the cell preparations analyzed in this study, CD203c was expressed exclusively by CD45^-^ non-hematopoietic cells. We have also previously observed a rare population of mast cells that express CD203c in the fetal liver, but only in cell preparations greatly enriched in leukocytes and depleted of hepatocytic cells (38). Seo et al. observed CD203c gene expression in the livers of fetal dairy calves, but which cell type expressed CD203c mRNA was not determined (50). They also noted a rapid decline in CD203c gene expression after birth. In the adult human liver, Yano et al. observed CD203c expressed by hepatocytes and in the portal area and on the apical plasma membrane of bile duct cells with higher staining in neoplastic bile duct cells than observed in surrounding normal tissues (51).

The expression of CD203c on fetal hepatoblasts was confirmed in a number of ways. CD203c^+^ cells expressed high levels of CD326 and comprise mostly large and complex cells based on flow cytometric light scatter characteristics. Previous gene expression analyses of CD14^low^CD326^++^ cells, which largely overlap with the CD203c^+^ cell population, showed that this cell population comprised the population of fetal liver cells that express albumin, CK19, *c-Met*, α-fetoprotein, hepatic nuclear factor 3α, prospero-related homeobox 1, cytochrome P450 3A7, α_1_-antitrypsin, and transferrin (9). These findings are also in line with past observations that CD326 is a marker of fetal hepatoblast and hepatocytic stem cells (3, 4, 7), but made the distinction that cells expressing lower levels of CD326 – and no CD203c – do not express these hepatoblast markers. Cell surface expression of recognized hepatocyte antigens such as CD24, CD26, and CD49f (9), further confirm CD14^low^CD203c^+^CD326^++^ cells to be hepatoblasts.

Low levels of CD203c expression were detected in the fetal liver parenchyma by microscopy, similar to what is observed with CD326. However, there were some notable differences between these two markers. Intense CD326 expression was observed on cholangiocytes, whereas no CD203c expression could be discerned on these cells. The more intense staining of CD326 observed surrounding vessels and the portal tract was also not apparent with CD203c. Thus, CD326 is a better marker at distinguishing subpopulations of hepatocytic cells by immunofluorescence microscopy. Cultured CD203c^+^CD326^++^ cells had the morphological appearance of hepatocytes and expressed albumin, CK8/18, and CK19. Co-expression of albumin and CK-19, a characteristic of hepatoblasts, was observed as well as a polarized expression of these markers indicative of differentiation. The expression of CD203c and CD326 was also examined after culture by both microscopy and flow cytometry. Cells lost CD203c expression rapidly in culture, an effect also observed, but to a lesser extent, with CD326. It is not unusual for fetal hepatoblast or adult hepatocyte gene-expression to be affected by culture with decreased expression often observed for metabolic and secretary gene products but increased expression of CK genes (9, 52–55).

Examination of hepatoblastoma cell lines and tumors confirmed that CD203c expression is found on at least some of these cells. Among the hepatoblastoma cell lines examined, CD203c was observed in association with CD326 expression - much like the normal expression of these markers on midgestation hepatoblasts. CD326, MYC, and CK19 expression have previously been associated with a poor prognosis for hepatoblastoma and is more often found on tumors described as embryonic in origin (56–58). It is worth pointing out that the cells expressing CD203c after extensive culture did so in contrast to the normal behavior of fetal hepatoblasts. Given the effects of culture and growth selection may have on gene expression by hepatoblastoma cell lines, it was important to examine CD203c expression on hepatoblastomas. In the several cases examined, CD203c expression was found in tumors also expressing CD326 and were associated with an embryonal histology. CK19 expression was, however, not observed in the tumor cells. The single fetal hepatoblastoma examined did not express CD203c or CD326. As the number of cases examined was minimal, further cases need to be examined to determine if CD203c expression offers any diagnostic insight.

In pediatric cancer, cell-of-origin can influence a persistent epigenetic state (59, 60). Our studies in the childhood muscle cancer rhabdomyosarcoma demonstrate that cell-of-origin can affect not only histological subtype but also therapeutic susceptibility (59, 61) and have identified the drug entinostat that silences the pathognomonic *PAX : FOXO* mutations in rhabdomyosarcoma. A broader question is whether phenocopy or cell-of-origin might give therapeutic insights in hepatoblastoma. As an example of examining cholangiocyte-precursor-like hepatoblastoma tumor cell markers, LIN28B, is known not only a hepatoblastoma marker but also key to hepatoblast maintenance/blockade of differentiation (62) and is targetable with the FDA-approved therapeutic DFMO (63). From a different approach, examination of normal human fetal hepatoblast markers by single cell analysis uncovers selective enrichment of both IGF2 and DLK1 (Delta Like Non-Canonical Notch Ligand 1 surface protein) – themselves targetable by clinically-investigated IGF1R inhibitors or antibody/CAR-T approaches, respectively. Thus, these lineage of origin studies open new opportunities for targeted therapies and accompanying biomarkers.

A number of other antigens were highlighted in our analyses of fetal liver cells that were either not previously recognized to be expressed by fetal hepatoblasts or hepatocytes at any stage of ontogeny. A number of these, such as CD63, CD73, CD99, CD146, and SSEA-4, are worthy of further investigation as they may distinguish subsets of CD326^++^ hepatoblasts based on their differential staining of the hepatoblast population. The tetraspanin CD63 is involved with exosome formation and plays an important role in viral infections such as hepatitis B infection (64). CD63 also interacts with CD82, another tetraspanin protein that was expressed by the fetal hepatoblasts (28). CD99 is a widely expressed protein that has been associated with many cell functions including adhesion and migration (34). It has been found highly expressed in a number of different tumors including hepatocellular carcinomas (65). CD146 is an adhesion molecule that is also expressed on hepatocellular carcinomas (66). SSEA-4 is a glycosphingolipid antigen previously observed by Dan et al. to be expressed by cultured fetal liver progenitors (4). Staining for SSEA-4 resulted in notably distinct positive and negative populations of CD326^++^ hepatoblasts.

The remaining antigens highlighted in the screening of fetal liver cells had a mostly homogeneous expression pattern on CD326^++^ hepatoblasts. This includes CD58, which has been observed on hepatocytic cell lines and in chronic hepatitis (67, 68). CD112 and CD166 that were observed by El Kehdy et al. to be expressed on cultured adult liver stem cells (69). The metalloproteinase CD156c and the immune checkpoint molecule CD276 are additional antigens whose expression is shared by fetal hepatoblasts and hepatocellular carcinomas (70–73). CD276 was also highly expressed by the four hepatoblastoma cell lines analyzed. Lastly, CD298 was only modestly expressed on hepatoblasts, with higher expression on LSECs. Previously expression of this ion transport molecule was reported in rat liver tissue with lower levels observed in young rats (74). The roles of these various cell surface molecules in the function of fetal hepatoblasts remain to be fully elucidated.

In conclusion, a flow cytometry based approach was used to screen human fetal liver cells for over 300 cell-surface antigens to identify those useful in discriminating among the many cell populations that comprise the developing liver. A more complete understanding of the phenotypic profiles of fetal hepatoblasts, LSECs, and hematopoietic cells is presented. A closer focus on hepatoblasts identified many widely expressed antigens as well antigens with differential expression that may be useful in identifying different stages of differentiation, maturation, or functional diversity. CD203c was a newly identified marker of fetal hepatoblasts with broad expression that was restricted to the hepatoblast population, save for its expression on mast cells and basophils. CD203c expression was found to be a marker of some hepatoblastomas using cell lines and histochemical analysis of tumors.

## Materials and methods

### Human fetal liver tissues

Human midgestation livers were obtained with approval of the University of California at San Francisco Institutional Review Board. Written informed consent was obtained by San Francisco General Hospital staff, not the authors of this report, for the donations from women undergoing elective abortions. Specimens were collected shortly after termination of the pregnancy and gestational age was determined based on the foot-length of the specimens. The sex of the specimens was not identified. Samples were kept on ice and transported to the laboratory in phosphate-buffered saline (PBS) or cell culture medium with antibiotics.

Biosafety level 2 procedures were used in handling human biological specimens in the laboratory. Cells were initially isolated by a method previously described (9, 20), but modifications occurred over the course of the study with changes to the availability and purity of the collagenase preparations. In the latest protocol iteration, liver tissue was minced and incubated with a mix of 1.0 mg/mL DE Collagenase 600 and DNase 100 KU/ml (Sigma-Aldrich) for 20 min at 37°C. After enzymatic dissociation, the tissue was homogenized further by pouring through a steel mesh and a 100 µm filter. Elimination of the major portion of red blood cells was achieved by incubation with unlabeled anti-CD235 antibody (antibodies used in this study are listed in **Supplemental Table S1**) and subsequent biomagnetic bead separation (Qiagen GmbH) (21).

### Flow cytometry

Analytical flow cytometry was performed as described in (21). Samples, containing freshly isolated or cultured cells, were stained with monoclonal antibodies labeled with fluorescent dyes listed in **Supplementary Table 1**. Cell sorting and additional phenotype acquisition was performed as previously described using a FACSAria flow cytometer (BD) (9).

### Immunofluorescence staining

Fetal liver sections were prepared, stained, and analyzed by epifluorescence microscopy as previously described (9). Briefly, livers were fixed with 10% formalin, processed through a sucrose gradient (10%, 20%, 30% and 50% in PBS) and frozen in Tissue-Tek Optimal Cutting Temperature compound (Ted Pella Inc., Redding, CA). Cryo-blocks were sectioned on a Cryostat Leica CM1850 UV (Leica Biosystems Nussloch GmbH, Nussloch, Germany). Staining was performed with primary and secondary antibodies. Nuclei were stained with 4′,6-diamidino-2-phenylindole (DAPI).

### Cell culture

Fetal liver cell cultures were performed as previously described (75). Cultures were grown in 6 wells plates with William’s E Medium (Gibco) and epidermal growth factor 0.2 ug/ml and Cell Maintenance Supplement Pack (#CM4000, Thermo Fisher Scientific) providing 0.1 uM dexamethasone, 6.25 ug/ml human recombinant insulin, 6.25 ug/ml human transferrin, 6.25 ng/ml selenous acid,1.25 mg/ml bovine serum albumin, 5.35 ug/ml linoleic acid, 2 mM GlutaMAX and 15 mM 4-(2-hydroxyethyl)-1-piperazineethanesulfonic acid. Cells were harvested with TrypLE Enzyme solution (Life Technologies), stained, and analyzed by flow cytometry.

### Hepatoblastoma cell lines and RNAseq data

Hepatoblastoma cell lines HB214, HB295, HB243, HB279, HB282 and HB284 were maintained as previously described (40). Additional descriptions of the tumor origins of the hepatoblastoma cell lines are presented in that publication. Briefly, pathology reports indicated that the main cellular components of the tumors from which these cell lines were derived are fetal (HB214, HB295), embryonal (HB243, HB282, HB284), and embryonal and macrotrabecular (HB279). HB-279 and HB-284 were obtained from the same patient at primary and recurrence resection. Raw FASTQ sequencing files were aggregated from sources described in Kats et al. Gene expression was quantified using STAR aligner with RSEM. Conventional RNAseq of hepatoblastoma datasets previously reported Kats et al. are available from the European Genome-Phenome (EGA) Repository as study EGAS00001004827 and dataset EGAD00001006621.

### Immunohistochemical staining of hepatoblastomas

After Institutional Review Board approval, representative formalin-fixed, paraffin embedded tissue blocks from three hepatoblastomas were retrieved from the pathology archives at Texas Children’s Hospital, and de-identified. Immunohistochemical stains for CD203c, CD326, and CK19 were performed on 5-micron thick, formalin-fixed, paraffin-embedded tumor tissue sections deparaffinized to water. Antigen retrieval was performed using citrate for 25 minutes, followed by primary antibody incubation at room temperature. Primary antibodies used were CD203c polyclonal antibody (**Supplementary Figure 1**; used at 1:200 dilution), CD326 (clone 323/A3; used at 1:200 dilution), or CK19 (clone RCK108; used at 1:2000 dilution). Bound primary antibody was detected using the Vector ImPRESS Goat anti Rabbit (MP-7451) and Goat anti-Mouse (MP-7421) kits.

### Data presentation and statistical analysis

Flow cytometry data were analyzed using FlowJo software, versions 9 and 10 (FlowJo, Inc.; Ashland, OR). Statistical analysis and charting were performed using Aabel NG software (Gigawiz Ltd. Co. OK, USA) and GraphPad Prism version 9.3.1(GraphPad Software, San Diego, California, USA), both for Macintosh.

## Data availability statement

The datasets presented in this study can be found in online repositories. The data presented in the study are deposited in the https://flowrepository.org repository, accession number id/FR-FCM-Z58Z.

## Ethics statement

The studies involving human participants were reviewed and approved by University of California at San Francisco Institutional Review Board. The patients/participants provided their written informed consent to participate in this study. De-identified hepatoblastoma specimens were studied with approval of the Institutional Review Board of Texas Children's Hospital.

## Author contributions

MM conceived of the study, contributed to data acquisition and interpretation, and drafted the manuscript. MF, AG, RG, CN, AB, and GO contributed to data acquisition and interpretation of studies presented in **Figures 1**-**7**. DL-T provided patient samples and performed immunohistochemical analyses of these samples (**Figure 9**). CK contributed to the design of hepatoblastoma experiments (**Figures 7** and **8**), data acquisition and interpretation, and drafting of the manuscript. DK and SC contributed cells and methods for hepatoblastoma studies. KM performed bioinformatic analysis. All authors contributed to the article and approved the submitted version.
