# Supplementary material for: CD203c is expressed by human fetal hepatoblasts and distinguishes subsets of hepatoblastoma
Source: Front Oncol. 2023 Feb 9;13:927852. doi: 10.3389/fonc.2023.927852 (PMC9947649; doi:10.3389/fonc.2023.927852)
Supplement: Supplementary file 1 [file Image_1.pdf]

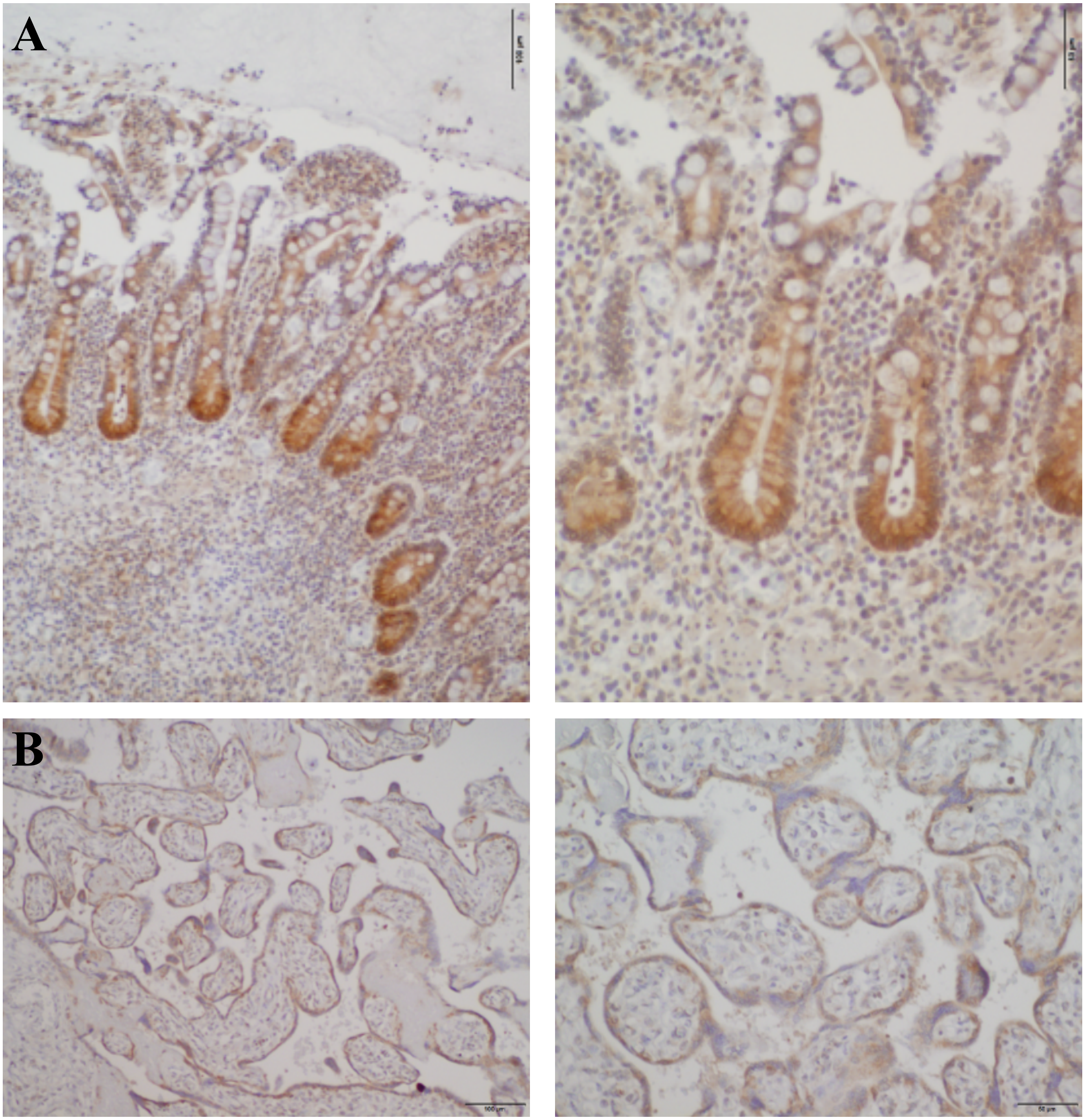

Supplementary Figure 1. CD203c polyclonal-antibody validation staining for immunohistochemistry. **(A)** CD203c (ThermoFisher Scientific, product #PA5-83680) staining is shown in the intestine under low power (left) and high power (right). 100µm (left) and 50µm (right) vertical scale bars are shown in the upper-right corner. **(B)** CD203c staining of placental tissue with low power (left) and high power (right) images. Horizontal 100µm (left) and 50µm (right) scale bars are shown in the lower-right.
