# Supplementary material for: CD203c is expressed by human fetal hepatoblasts and distinguishes subsets of hepatoblastoma
Source: Front Oncol. 2023 Feb 9;13:927852. doi: 10.3389/fonc.2023.927852 (PMC9947649; doi:10.3389/fonc.2023.927852)
Supplement: Supplementary file 2 [file Table_1.pdf]

Supplementary Table 1. List of antibodies used in this study.

Antibody	Fluorophore[1]	Company[2]	Clone	Isotype[3]
Antibodies recognizing human antigens				
CD9	PE	BioLegend	HI9a	IgG1
CD9	PE	BD Pharmingen	M-L13	IgG1
CD13	PE	BioLegend	WM15	IgG1
CD13	APC	BioLegend	WM15	IgG1
CD14	FITC	Invitrogen-Thermo Fisher Scientific	TüK4	IgG2a
CD14	FITC	BioLegend	HCD14	IgG1
CD14	PE-Cy7	BioLegend	HCD14	IgG1
CD24	PE	BioLegend	ML5	IgG2a
CD24	AlexaFluor 488	BioLegend	ML5	IgG2a
CD24	FITC	BioLegend	ML5	IgG2a
CD24	PE-Cy7	BioLegend	ML5	IgG2a
CD26	PE	BioLegend	BA5b	IgG2a
CD26	FITC	BioLegend	BA5b	IgG2a
CD26	APC	BioLegend	BA5b	IgG2a
CD29	APC	BioLegend	TS2/16	IgG1
CD34	PE	BioLegend	581	IgG1
CD36	PE	BioLegend	5-271	IgG2a
CD45	FITC	BioLegend	HI30	IgG1
CD46	PE	BioLegend	TRA-2-10	IgG1
CD49a	PE	BioLegend	TS2/7	IgG1
CD49e	PE	BioLegend	NKI-SAM-1	IgG2b
CD49f	PE	BioLegend	GoH3	rat IgG2a
CD58	PE	BioLegend	TS2/9	IgG1
CD63	PE	BioLegend	H5C6	IgG1
CD73	PE	BioLegend	AD2	IgG1
CD82	PE	BioLegend	ASL-24	IgG1
CD99	PE	BioLegend	HCD99	IgG2a
CD112	PE	BioLegend	TX31	IgG1
CD146	PE	BioLegend	SHM-57	IgG2a
CD146	PE	R&D Systems	128018	IgG1
CD146	FITC	R&D Systems	128018	IgG1
CD156c	PE	BioLegend	SHM-14	IgG1
CD166	PE	BioLegend	3A6	IgG1
CD203c	PE	Beckman Coulter	97A6	IgG1
CD203c	PE	Miltenyi Biotec	FR3-16A11	IgG1
CD203c	PE	BioLegend	NP4D6	IgG1
CD203c		Thermo Fisher Scientific		Polyclonal
CD235a		American Type Culture Collection	10F7MN	IgG1
CD235a		Bio-X-Cell	6A7M	IgG1
CD235a	FITC	BioLegend	HI264	IgG2a
CD276	PE	BioLegend	MIH42	IgG1
CD298	PE	BioLegend	LNH-94	IgG1
CD324	PE	BioLegend	67A4	IgG1
CD326	APC	Miltenyi Biotec	HEA-125	IgG1
CD326	AlexaFluor 488	BioLegend	9C4	IgG2b
CD326	APC	BioLegend	9C4	IgG2b
CD326		Thermo Fisher Scientific	323/A3	IgG1
Cytokeratin 19		DAKO	RCK108	IgG1
SSEA-4	PE	BioLegend	MC-813-70	IgG3
Non-specific mouse antibodies used as isotype controls				
IgG1	FITC	BioLegend	MOPC-21	
IgG2a	FITC	BioLegend	MOPC-173	
IgG1	APC	BioLegend	MOPC-21	
IgG1	PE	BioLegend	MOPC-21	
IgG3	PE			
IgG1	PE-Cy7	BioLegend	MOPC-21	
IgG1	FITC	Invitrogen-Thermo Fisher Scientific		Polyclonal
IgG2a	FITC	Invitrogen-Thermo Fisher Scientific		Polyclonal
IgG1	PE	Invitrogen-Thermo Fisher Scientific		Polyclonal
IgG2a	PE	Invitrogen-Thermo Fisher Scientific		Polyclonal
IgG2b	PE	Invitrogen-Thermo Fisher Scientific		Polyclonal
IgM	PE	Invitrogen-Thermo Fisher Scientific		Polyclonal
IgG1	APC	Invitrogen-Thermo Fisher Scientific		Polyclonal
Antibodies recognizing mouse antibodies				
Goat anti-mouse IgG1	AlexaFluor 568	Invitrogen-Thermo Fisher Scientific		Polyclonal
Goat anti-mouse IgG2a	AlexaFluor 568	Invitrogen-Thermo Fisher Scientific		Polyclonal
Goat anti-mouse IgG2b	AlexaFluor 568	Invitrogen-Thermo Fisher Scientific		Polyclonal
